# IgM antibodies against phosphorylcholine measured early after acute ST-elevation myocardial infarction in relation to atherosclerotic disease burden and long-term clinical outcome

**DOI:** 10.1371/journal.pone.0215640

**Published:** 2019-04-19

**Authors:** Eva Cecilie Knudsen, Ingebjørg Seljeflot, Tonje Amb Aksnes, Jan Eritsland, Harald Arnesen, Geir Øystein Andersen

**Affiliations:** 1 Department of Cardiology, Oslo University Hospital, Oslo, Norway; 2 Center for Clinical Heart Research, Oslo University Hospital, Oslo, Norway; 3 Faculty of Medicine, University of Oslo, Oslo, Norway; 4 Center for Heart Failure Research, Oslo University Hospital, Oslo, Norway; 5 Section for Interventional Cardiology, Heart-, lung-, and Vascular-Disease Clinic and Section of Cardiovascular and Renal Research, Oslo University Hospital, Oslo, Norway; Niigata Daigaku, JAPAN

## Abstract

**Purpose:**

Studies have reported an association between low levels of natural immunoglobulin M antibodies against phosphorylcholine(IgM anti-PC) and worse prognosis in patients with coronary artery disease (CAD). The aims of the present study were, in patients with ST-elevation myocardial infarction (STEMI); 1) to compare serum levels of IgM anti-PC measured acutely and after 3 months; 2) to study an association between levels of IgM anti-PC and the severity ofCAD, and; 3) to investigate whether IgM anti-PC levels are associated with long-term clinical outcome.

**Methods:**

A total of 213 patients without known diabetes (median age 59 years) with a PCI treated STEMI were enrolled. IgM anti-PC was measured in-hospital and after 3 months. Median follow-up time was 6.5 years (all-cause mortality, non-fatal myocardial re-infarction, recurrent ischemia causing hospital admission, heart failure and stroke). The severity of CAD was evaluated by coronary angiograms and patients were classified as having single- or multi-vessel disease and by SYNTAX score (SXscore).

**Results:**

IgM anti-PC levels were stable over time when measured acutely and after 3 months. Patients with multi-vessel disease and high SXscore had significantly lower levels of IgM anti-PC in the acute phase of STEMI. Low levels of IgM anti-PC (the 25 percentile) measured acutely were associated with a 2-fold increase in the odds of having multi-vessel disease (adjusted OR 2.28 (95% CI 1.17, 4.44), p = 0.016), but not with high SXscore (Crude OR 2.20 (95% CI 0.96, 5.07), p = 0.06). Fifty-three patients experienced a new clinical event during long-term follow-up. Low levels of IgM anti PC were not associated with worse prognosis, (crude HR 1.54 (0.87–2.76), p = 0.14).

**Conclusion:**

STEMI patients with multi-vessel disease or high SXscore had significantly lower levels of IgM anti-PC in the acute phase and low levels were associated with multi-vessel disease, but not with worse clinical outcome during long-term follow-up.

## Introduction

The atherosclerotic process leading to plaque rupture and coronary occlusion is the leading underlying cause of myocardial infarction and sudden cardiac death, and atherosclerosis is considered at least partly to be an inflammatory disease [1–3]. Autoantibodies directed towards a variety of oxidation-specific epitopes or self—antigens have been identified as mediators in this complex inflammatory environment [4]. Oxidation-specific epitopes belong to a class of danger-associated molecular patterns (DAMPS), and DAMPs are recognized by pattern recognition receptors (PRR) on macrophages, natural antibodies and other effector proteins from the innate immune system [5]. Phosphorylcholine (PC) is a known oxidation-specific epitope on oxidized low-density lipoprotein (OxLDL) and natural immunoglobulin M (IgM) antibodies against PC (IgM anti-PC) may have atheroprotective properties in response to oxidative stress [4]. Low levels of IgM anti-PC have been shown, in some studies, to be associated with worse prognosis in patients with acute coronary syndrome [6]. However, little is known about the levels of IgM anti-PC during an acute ST-elevation myocardial infarction (STEMI), how levels change in patients post-MI, or a possible association between levels of IgM anti-PC measured acutely and the extent of coronary artery disease, or new clinical events after STEMI. The aims of the present study were therefore; in patients with STEMI 1) to compare serum levels of IgM anti-PC measured acutely with measurements after 3 months; 2) to study a possible association between levels of IgM anti-PC and the severity of coronary artery disease, and; 3) to investigate whether IgM anti-PC levels are associated with long-term clinical outcome.

## Methods

### Study population

A total of 213 patients with a primary percutaneous coronary intervention (PCI) treated STEMI, originally included in a prospective observational cohort study on the incidence of abnormal glucose regulation classified by an oral glucose tolerance test (OGTT) were investigated [7]. Patients with acute STEMI admitted to the Coronary Intensive Care Unit, Oslo University Hospital, Ullevål, Oslo, Norway, were prospectively enrolled from November 2005 to May 2007. The cohort is described in detail elsewhere [7]. In brief, patients with a PCI treated STEMI without known diabetes were included if they were hemodynamically stable, without chest pain, persistent hyperglycemia, nausea, age <85 years and with serum creatinine <200 μmol/L. Patients with cardiac arrest or cardiogenic shock, or patients unwilling to give informed consent, were excluded. The total number of STEMI patients in the inclusion period was 2251 and 213 patients (9.5%) were included. STEMI was defined according to the universal definition of myocardial infarction as typical rise and fall of the cardiac biomarker troponin T with at least one value above the 99th percentile of the upper reference limit, in patients presenting with symptoms of ischemia together with new ST elevation at the J-point in two contiguous leads with the cut-off points: 0.2 mV in men or 0.15 mV in women in leads V2–V3 and/or 0.1 mV in other leads *or* new left bundle-branch block [8]. The extent and complexity of coronary artery disease was evaluated by coronary angiograms and patients were classified in two ways, as single- or multi-vessel disease, and by the SYNTAX score (SXscore). Single- or multi-vessel diseases were defined as the presence of one or more major coronary arteries with at least > 50% intraluminal diameter narrowing. SXscore for each patient was calculated retrospectively by a re-evaluation of the coronary angiograms by scoring all coronary lesions with a stenosis >50% in vessels larger than 1.5 mm using a SXscore online calculator [9]. Two independent cardiologists evaluated all variables involved in the calculation. In the event of disagreement, the final decision was made by consensus. Low SXscore was defined as ≤ 22, an intermediate score as 23 to 32, and a high score as ≥33 [10]. In the present study, SXscore was dichotomised into low or high score ≤22 or >22.

The protocol with potential sub-studies was approved by the Norwegian Regional Committee for Medical Research Ethics, South-East Norway, on May 19, 2005. This sub-study was conducted according to the Declaration of Helsinki and all patients gave written informed consent to participate in the study. The main study is registered at www.clinicaltrials.gov, NTC00926133.

### Laboratory methods

Blood samples were collected between 8 and 10 am after an overnight fast at median time 16.5 hours after primary PCI and repeated after 3 months. Blood samples were separated within 1 hour by centrifugation at 2500g for 10 min and stored at—80°C until analyzed. Serum cardiac specific Troponin T (TnT) was measured by electrochemiluminescence technology for quantitative measurement (3^rd^ generation TnT, Elecsys 2010, Roche, Mannheim, Germany). Detection of IgM anti-PC (U/mL) was performed in serum samples by an enzyme-linked immunosorbent assay (ELISA) method (CVDefine, Athera Biotechnologies, Stockholm, Sweden). The lower detection limit of the assay is 0.5 U/ml. Inter-assay coefficient of correlation was <10%.

### Long-term follow-up

After a median follow-up time of 6.5 years, all patients were contacted and interviewed by telephone, regarding their health status and any hospital admissions. Closing date was set to March13th 2013. In case of re-hospitalization, hospital records were collected. The primary endpoint was defined as the composite of all-cause mortality, myocardial re-infarction, re-hospitalization for unstable angina, ischemic stroke, and heart failure, whichever occurred first.

### Statistical analyses

Parametric and non-parametric statistics were used. A p-value <0.05 was considered statistically significant. Continuous data were expressed as median (25^th^, 75^th^ percentiles) or means (± SD), and categorical data as frequencies (%). Comparison between two groups was assessed by the Mann-Whitney test for continuous variables and the Chi square test for categorical data. Comparison between two related samples was assessed by the Wilcoxon test.

The continuous variable IgM anti-PC was dichotomize into low and high levels at the 25 percentile based on linear trend analysis of the association between quartiles of IgM anti-PC and the presence of multi-vessel disease.

Multivariable logistic regression analyses were performed adjusting for clinical covariates. Potential confounders that were associated with both multi-vessel disease and IgM anti-PC with a p-value <0.2, were included in the model. The following risk factors were included in the final model; age, hypertension, smoking and, gender, in addition to IgM anti-PC. Univariate analyses of this dynamic cohort with censored data (Kaplan-Meier survival, Log-Rank test and Cox proportional hazard models) were performed.

Receiver operator characteristic (ROC) curve analysis with the corresponding area under the curve (AUC) with 95% confidence interval was performed to determine the overall performance of IgM anti-PC to predict clinical outcome.

The STROBE guidelines for cohort studies were followed [11]. Analyses were performed using SPSS software, 2015, version 23 (SPSS, Chicago, L).

## Results

### Baseline characteristics

A total of 213 patients with median age 59 (52, 68) years were enrolled. A majority of the patients were men, half of them were smokers, and hypertension was common.

Clinical characteristics of the study population according to low or high level of IgM anti-PC measured acutely are shown in Table 1.

**Table 1:** Clinical characteristics related to IgM anti-PC levels measured in-hospital.

Variables	IgM anti-PC* ≤24,6 U/ml (n = 53)	IgM anti-PC* >24,6 U/ml (n = 160)	P value
Age (years)	60 (53.5, 70.5)	58 (51.5, 66.5)	0.09
Male	40 (75.5)	135 (84.4)	0.1
Previous disorder:			
Treated hypertension	10 (18.9)	47 (29.4)	0.09
Myocardial infarction	5 (9.4)	8 (5)	0.2
Angina pectoris	3 (5.7)	3 (1.9)	0.2
Treated hyperlipidaemia	7 (13.2)	12 (7.5)	0.2
Status at baseline			
Current smoker	25 (47.2)	78 (48.6)	0.5
Multi-vessel disease	27 (50.9)	54 (33.8)	0.02
Waist circumference (cm)	100 (95, 108)	100 (94, 107)	0.6
BMI (kg/m2)	26.5 (24.4, 28.4)	26 (24.4, 28.7)	0.6
Cholesterol (mmol/l)	4.8 (4.3, 5.8)	5.2 (4.6, 5.9)	0.2
LDL (mmol/l)	3.20 (2.77, 3.87)	3.43 (2.76, 4.15)	0.2
C reactive protein (mg/l)	11.6 (7.4, 33.9)	12.1 (6.1, 33.0)	0.8
scTnT _peak value_ (ug/l)	4.3 (2.4, 8.7)	4.8 (2.5, 9.1)	0.5
Stent in culprit lesion	52 (98.1)	152 (95.0)	0.3
Gp IIb/IIIa antagonist treated	18 (34)	55 (34.4)	0.6
Time from symptoms to balloon (min)	234 (143, 415)	218 (136.5, 396.0)	0.6
Medication at hospital discharge			
Aspirin	53 (100)	160 (100)	
Clopidogrel	53 (100)	158 (98.8)	0.6
β-blockers	37 (69.8)	136 (85.0)	0.01
Statins	52 (98.1)	158 (98.8)	0.6
Angiotensin converting enzyme-inhibitors	4 (7.5)	26 (16.3)	0.08
Angiotensin II-receptor blockers	5 (9.4)	13 (8.1)	0.5

Data are presented as median with inter-quartile range or proportions.

*Above or below 25 percentile.

BMI: body mass index, scTnT: serum-c TroponinT, LDL: low-density lipoprotein, GP:glycoprotein

### Changes in the level of IgM anti-PC measured in-hospital and after 3 months

To evaluate a possible change in the level of IgM anti-PC from the acute phase to a stable condition, IgM anti-PC measures were repeated in 200 patients after 3 months. The mean level of IgM anti-PC measured in-hospital and at follow-up was 50.39 (37.25) U/ml and 52.37 (38.46) U/ml, respectively. The mean change of IgM anti-PC was 1.98 (14.21) U/ml, which was not statistically significant, p = 0.51.

### Association between low levels of IgM anti-PC and severeity of coronary artery disease

Clinical characteristics of the study population related to the extent of coronary artery disease are summarized in Table 2. Patients with severe coronary artery disease defined as multi-vessel disease or by high SXscore, were older and had significantly lower levels of IgM anti-PC measured in-hospital. When dividing IgM anti-PC levels into quartiles, there was a significant trend across the quartiles for the presence of multi-vessel disease with decreased levels of IgM anti-PC (p = 0.02), identifying a threshold for IgM anti-PC at the 25 percentiles, 24.6 U/ml, thus IgM anti-PC were divided into high and low levels at the 25 percentiles. Low levels of IgM anti-PC (≤24.6 U/ml) measured acutely were associated with a 2-fold increase in the odds of having multi-vessel disease (crude OR 2.04 (95% CI 1.09, 3.83), p = 0.03). After adjusting for gender; smoke, and age (age dichotomized above and below 67 years), the association remained significant (adjusted OR 2.28 (1.17, 4.44), p = 0.016). Low levels of IgM anti-PC (≤24.6 U/ml, 25 percentile) measured acutely were not significantly associated with high SXscore. Crude OR 2.20 (95% CI 0.96, 5.07), p = 0.06.

**Table 2:** Clinical characteristics related to the extent of coronary artery disease diagnosed in-hospital.

	Single-vessel disease (n = 132)	Multi-vessel disease (n = 81)	P value
Anti-PC (U/ml)	43.4 (26.6, 67.1)	32.7 (21.5, 52.6)	0.03
Age (years)	57 (51, 63.5)	63 (54.5, 72)	0.003
Male	103 (78.3)	72 (88.8)	0.03
Current smoker	72 (54.6)	31 (38.3)	0.02
Treated hypertension	33 (25)	24 (29.6)	0.3
Previous myocardial infarction	7 (5.3)	6 (7.4)	0.4
BMI (kg/m^2^)	26.3 (24.4, 28.7)	26 (24.6, 28.1)	0.5
Cholesterol (mmol/l)	5.2 (4.6, 5.8)	5.1 (4.5, 5.8)	0.5
OxLDL (Um/l)	58.5 (47.2, 71.1)	55.7 (48.7, 71.8)	0.6
C-reactive protein (mg/l)	11.99 (6.23, 33.01)	11.78 (7.37, 32.96)	0.9
	Low SXscore (n = 185)	High SXscore (n = 28)	P value
Anti-PC (U/ml)	40.9 (26.1, 67.8)	26.7 (18.7, 44.5)	0.01
Age (years)	57 (51, 65.5)	68 (60, 75)	<0.001
Male	152 (82.2)	23 (82.1)	0.6
Current smoker	91 (49.2)	12 (42.9)	0.3

Data are presented as median with interquartile range or proportions.

BMI: body mass index, oxLDL: oxidized low-density lipoprotein

### Association between low levels of IgM anti-PC and clinical outcome

The median follow-up time for clinical events was 6.5 (5.9, 7.0) years. Of the total population (n = 213), 53 (24.9%) patients experienced a new clinical event, including 13 patients with recurrent ischemia causing hospital admission, 9 patients died, 1 of myocardial re-infarction, 1 of cancer, 3 of an infectious disease with multi-organ failure, and 4 died of unknown reason, 3 patients underwent a non-fatal stroke, 4 patients were diagnosed with heart failure, and 24 patients experienced a non-fatal re-infarction. No significance difference was found between IgM anti-PC as a continuous variable and worse clinical outcome when quantified by ROC-curve analysis, AUC 0.56, 95% CI 0.47–0.65, p = 0.19. When IgM anti-PC were dichotomized into low and high levels, the probability of remaining free from a new clinical event did not differ between the groups (Log-Rank, p = 0.14), (crude HR 1.54 (95% CI 0.87–2.76), p = 0.14).

In an additional analysis the primary endpoint was defined as the composite of cardiac mortality, myocardial re-infarction, re-hospitalization for unstable angina and ischemic stroke (n = 205 patients). The probability of remaining free from a new clinical event between the groups remained non-significant (Log-Rank, p = 0.40), (crude HR 1.31 (95% CI 0.70–2.46), p = 0.40.

## Discussion

In this cohort of STEMI patients the levels of IgM anti-PC were stable over time when measured during acute illness in-hospital and again in a stable condition after 3 months. Patients with advanced coronary heart disease defined as multi-vessel disease or high SxScore had significantly lower levels of IgM anti-PC during the acute STEMI. There was a significant association between low levels of IgM anti-PC and multi-vessel disease, but not with high SXscore or long-term clinical outcome.

The existence of IgM anti-PC in humans has been known for decades, but how levels of IgM anti-PC are regulated and their clinical relevance are not completely understood [4]. Some data indicate that IgM anti-PC are generated in the absence of known antigen stimulation by a specialized set of innate B cells, the B1cells [12]. B1 cells have been measured both in umbilical cord blood and adult peripheral blood, and the fraction of B1 cells have been shown to decline with age in normal individuals, which may contribute to disease progression in older age [12]. Furthermore, the levels of IgM anti-PC seem to vary between individuals at the same age [6]. In our study the levels of IgM anti-PC were stable over time when measured the first day after STEMI and again 3 months later. This result is in contrast to another study showing that levels of IgM anti-PC increased from day 1 to day 90 in a larger cohort with non-ST-elevation myocardial infarction (NSTEMI) and STEMI, discussed to be due to consumption of IgM anti-PC during the event [6]. The levels of IgM anti-PC were found to be significantly higher in a healthy population from New Guinea, where cardiovascular disease is almost unknown, as compared to Swedish controls, and among both cohorts, men had significantly lower levels of IgM anti-PC compared to women [13]. In another Swedish study the intra-individual serum levels of IgM anti-PC were stable over a four year period in 226 hypertensive patients, mean age 57.7 years [14]. These results indicate that the levels of IgM anti-PC vary between individuals, according to gender, potential life-style factors and underlying genetic variations, and seem to remain fairly stable over time in each individual. Clinical and experimental studies indicate that levels of IgM anti-PC are inversely correlated with cardiovascular disease progression [15] and that IgM anti-PC may mediate atheroprotection by neutralizing the proinflammatory properties of oxLDL [4, 5]. One of the major components of the atherosclerotic lesion is oxidized lipids such as oxLDL, and oxidization of LDL is known to facilitate the uptake of LDL in macrophages [4]. PC is a known oxidation-specific epitope on oxLDL and binding of IgM anti-PC to PC on oxLDL is proposed to block macrophage uptake by inhibiting the scavenger receptor and thus inhibit the foam cell formation [4].

In the present study, low levels of IgM anti-PC were associated with the severity of the atherosclerotic process defined as multi-vessel disease. This association indicate that low levels of IgM anti-PC measured early after acute STEMI are probably not related to the severity of myocardial necrosis or systemic inflammation, but rather, in line with previous data, a marker of high-risk patients with advanced atherosclerotic disease burden with reduced atheroprotective properties [6]. A growing body of evidence suggest that immunization against epitopes on oxLDL reduces experimental atherosclerosis, indicating that vaccination against atherosclerosis can be a new target of treatment in the future [16].

The SXscore was initially tested in patients with stable coronary artery disease, multi-vessel disease or complex coronary lesions allocated to PCI or coronary artery bypass graft (CABG) surgery in the landmark SYNTAX trial. However, several studies have also investigated the prognostic utility of the SXscore in patients with STEMI [17].

In the present study, patients with high SXscore had significantly lower levels of IgM anti-PC compared to patients with low SXscore. Only 28 patients were classified with high SXscore and probably due to lack of power we could not demonstrate a significant association between low levels of IgM anti-PC at baseline and the presence of high SXscore.

In this study low levels of IgM anti-PC were not associated with clinical outcome in patients with acute STEMI. The neutral results could be explained by a relatively young study population who experienced few clinical events during follow up, indicating that a larger number of patients have to be studied to detect an increased risk related to low levels of IgM anti-PC. Previous larger studies have reported that low levels of IgM anti-PC are associated with poor clinical outcome in patients with acute coronary syndromes [6], supporting the theory that circulating immune complexes of IgM anti-PC and PC may have atheroprotective properties [4].

The present study has certain limitations. Patients with known diabetes were excluded and possible associations between diabetes and inflammation could not be investigated. There were relatively few clinical endpoints, reflecting a low-risk patient group, indicating a lack of statistical power. The association between low levels of IgM anti-PC and outcome in patients with STEMI should be further investigated in forthcoming studies including a larger number of patients with long-term follow-up in order to elucidate IgM anti-PC as a possible prognostic marker in patients with acute STEMI. Further studies are also needed to determine the pathophysiological role of IgM anti-PC in relation to the development of atherosclerotic diseases, and to study IgM anti-PC as a possible risk marker in both primary and secondary prevention.

## Conclusions

The levels of IgM anti-PC were stable over time when measured acutely and again after 3 months in this cohort of STEMI patients. Patients with multi-vessel disease or high SXscore had significantly lower levels of IgM anti-PC in the acute phase of STEMI. Low levels of IgM anti-PC were also associated with multi-vessel disease after adjustment in multivariate analyses, indicating that IgM anti-PC are a marker of patients with advanced atherosclerotic disease burden. Low levels of IgM anti-PC were not associated with worse clinical outcome during long-term follow-up, although the study may lack statistical power to detect an increased risk due to the excellent clinical outcome.

## Supporting information

S1 File Main study dataset. (XLSX)

S2 File Dataset including the levels of IgM anti-PC measured in-hospital and after 3 months. (XLSX)

S3 File Study protocol. (DOC)
