# Supplementary material for: IgM antibodies against phosphorylcholine measured early after acute ST-elevation myocardial infarction in relation to atherosclerotic disease burden and long-term clinical outcome
Source: PLoS One. 2019 Apr 19;14(4):e0215640. doi: 10.1371/journal.pone.0215640 (PMC6474742; doi:10.1371/journal.pone.0215640)
Supplement: S3 File — (DOC) [file pone.0215640.s003.doc]

**CLINICAL STUDY PROTOCOL**

**Title**: Abnormal glucose REGULATION IN PATIENTS WITH STABLE CORONARY ARTERY DISEASE.

**Responsible for the study**: Department of Cardiology, OsloUniversity Hospital, Ullevål.

**Project leader**: Eva Cecilie Knudsen, MD, PhD. Department of Cardiology, and Center for Clinical Heart Research, Oslo University Hospital, Ullevål.

**Design**: Cohort study, long-term follow-up of 224 patients with stable coronary artery disease with and without previously known abnormal glucose regulation.

**Study center**: Department of Cardiology, Ullevål University Hospital.

# INTRODUCTION

Cardiovascular diseases and type 2 diabetes are closely correlated [1]. The risk for cardiovascular diseases increases along a spectrum of blood glucose concentration already apparent at levels regarded as normal [2]. The combination of glucometabolic disturbances and cardiovascular diseases are common and markedly increases the morbidity and mortality. Several studies have shown that normal glucose regulation is less common than impaired glucose regulation and type 2 diabetes in patients with acute myocardial infarction. Additionally, glucometabolic disturbances are a strong risk factor for future cardiovascular events after an acute myocardial infarction. Based on these considerations the European guidelines on DM, prediabetes, and cardiovascular disease recommend that patients without known diabetes, but with established cardiovascular disease should be investigated with an oral glucose tolerance test (OGTT) [3]. However, the American Heart Association Diabetes Committee of the Council on Nutrition, Physical Activity, and Metabolism recommended that patients without known diabetes, but with hyperglycemia during an episode of an acute coronary syndrome should be further evaluated to determine the severity of their metabolic derangements do not encourage routine use of OGTT after an acute coronary syndrome or myocardial infarction (9).

However, there exists no consensus about the timing of an OGTT after acute MI and the OGTT has also been shown to have poor reproducibility.

In the meantime HbA1c have been introduced as a diagnostic tool in

The pathophysiological mechanisms that explain the relationship between glucometabolic disturbances and cardiovascular diseases are not fully understood [1]. However, inflammation has been suggested to be one of the bridging link between abnormalities in glucose metabolism and atherosclerotic disorders [4]

Previously, we have studied the prevalence of impaired glucose regulation and type 2 diabetes in a Norwegian cohort of patients with a primary PCI treated STEMI without previously known diabetes. The cohort was further followed for new clinical events for a median time of 33 months after the acute STEMI. This cohort was included from November 2005 until May 2007.

Next year, about seven years later we want to repeat the prevalence and outcome study in the same cohort.

**AIMS OF THE STUDY**

**Primary objectives**:

1. To repeat the prevalence study of impaired glucose regulation and type 2 diabetes in a Norwegian cohort who underwent an acute ST-elevation myocardial infarction and performed an OGTT in the period of November 2005 until May 2007.
2. Validate the results of an OGTT performed very early after myocardial infarction, by repeating the test several years later.
3. Validate HbA1c as a diagnostic tool by comparing the test with the results of an OGTT performed at the same time-point in patients with stable coronary artery disease without previously known diabetes.
4. Study the relationship between abnormal glucose regulation diagnosed about 7 years after STEMI and long-term prognosis.
5. Contribute to an increased focus on undiagnosed abnormal glucose regulation in patients with stable coronary artery disease in Norway

**Secondary objectives**:

1. To study the time course of diabetes development in patients with known impaired glucose regulation and coronary artery disease.
2. To elucidate possible interactions between biomarkers of inflammation and coagulation, and the glucometabolic status in patients with stable coronary artery disease.
3. Investigate how physicians follow patients with stable coronary artery disease and known glucometabolic state ”in real-life”.

# Patient population:

Patients with a primary PCI treated STEMI hospitalized at the coronary care unit at Oslo University Hospital, Ullevål from November 2005 to May 2007. The patients were included after informed consent.

**The originally inclusion criterions were:**

- Acute ST-segment elevation infarction defined from ECG.
- All the patients have been treated by primary percutaneous coronary intervention in a defined culprit leasen.
- Stable patient without chest pain, nausea or heart failure.

**The originally exclusion criterions were:**

- Age> 85 years
- Known type 2 diabetes
- Unstable patient
- On-going chest pain and nausea
- On-going infusions
- Heart failure symptoms
- Serious kidney disease (Kreatinin>200 umol/l)

**Laboratory measurements**:

OGTT: A standardized oral glucose tolerance test will be performed after an overnight fasting period of at least 8 hours. OGTT will be performed between 08:00-10:00 a.m., but not in patients with ongoing hyperglycaemia. Blood samples are taken for analysis of plasma glucose before and 2 hours after drinking 75 g glucose dissolved in 250 ml water.

**Biochemical analyses**: Blood samples will be frozen at –80^0^C. Samples will be taken before and after OGTT. A broad spectre of biomarkers and hormones will be analyzed including, insulin, C-peptide, **IL-5,** HsCRP, MCP-1, PAI-1act, tPAag, **IgM anti-PC, proBNP.**

**Long-term follow-up:** A clinical examination with ECG will be performed at our outpatient clinic.

In may 2013, all patients will be contacted by phone. A standardized questionnaire will be used. Death, cardiac death, myocardial infarction, stroke and hospitalization for heart disease will be noted and hospital records will be checked in addition to the National Death Registry.

# Sub-study

To study a possible association between levels of IgM anti-PC measured acutely and the severity of coronary artery disease in patients with a primary PCI treated STEMI without known diabetes.

Reference List

1 Stern MP. Diabetes and cardiovascular disease. The "common soil" hypothesis. *Diabetes* 1995 Apr;**44**(4):369-74.

2 Bartnik M, Norhammar A, Ryden L. Hyperglycaemia and cardiovascular disease. *J Intern Med* 2007 Aug;**262**(2):145-56.

3 Ryden L, Standl E, Bartnik M, et al. Guidelines on diabetes, pre-diabetes, and cardiovascular diseases: executive summary. The Task Force on Diabetes and Cardiovascular Diseases of the European Society of Cardiology (ESC) and of the European Association for the Study of Diabetes (EASD). *Eur Heart J* 2007 Jan;**28**(1):88-136.

4 Libby P. Inflammation and cardiovascular disease mechanisms. *Am J Clin Nutr* 2006 Feb;**83**(2):456S-60S.
